# The miR-182-5p/GPX4 Pathway Contributes to Sevoflurane-Induced Ototoxicity via Ferroptosis

**DOI:** 10.3390/ijms25126774

**Published:** 2024-06-20

**Authors:** Lin Jin, Xiaopei Yu, Xuehua Zhou, Gang Li, Wen Li, Yingzi He, Huawei Li, Xia Shen

**Affiliations:** 1 Department of Anesthesiology, Eye and ENT Hospital, Fudan University, Shanghai 200031, China; jinlinfudan@163.com (L.J.);; 2 ENT Institute and Department of Otorhinolaryngology, Eye and ENT Hospital, State Key Laboratory of Medical Neurobiology and MOE Frontiers Center for Brain Science, Fudan University, Shanghai 200031, China; 20111260030@fudan.edu.cn (X.Y.); 09111010066@fudan.edu.cn (Y.H.); 3 NHC Key Laboratory of Hearing Medicine, Fudan University, Shanghai 200031, China; 4 Department of Ophthalmology and Vision Science, Eye and ENT Hospital, Shanghai Medical College, Fudan University, Shanghai 200031, China

**Keywords:** sevoflurane, ototoxicity, ferroptosis, miR-182-5p, GPX4

## Abstract

Our study aimed to investigate the role of ferroptosis in sevoflurane-induced hearing impairment and explore the mechanism of the microRNA-182-5p (miR-182-5p)/Glutathione Peroxidase 4 (GPX4) pathway in sevoflurane-induced ototoxicity. Immunofluorescence staining was performed using myosin 7a and CtBP2. Cell viability was assessed using the CCK-8 kit. Fe^2+^ concentration was measured using FerroOrange and Mi-to-FerroGreen fluorescent probes. The lipid peroxide level was assessed using BODIPY 581/591 C11 and MitoSOX fluorescent probes. The auditory brainstem response (ABR) test was conducted to evaluate the hearing status. Bioinformatics tools and dual luciferase gene reporter analysis were used to confirm the direct targeting of miR-182-5p on GPX4 mRNA. GPX4 and miR-182-5p expression in cells was assessed by qRT-PCR and Western blot. Ferrostatin-1 (Fer-1) pretreatment significantly improved hearing impairment and damage to ribbon synapses in mice caused by sevoflurane exposure. Immunofluorescence staining revealed that Fer-1 pretreatment reduced intracellular and mitochondrial iron overload, as well as lipid peroxide accumulation. Our findings indicated that miR-182-5p was upregulated in sevoflurane-exposed HEI-OC1 cells, and miR-182-5p regulated GPX4 expression by binding to the 3′UTR of GPX4 mRNA. The inhibition of miR-182-5p attenuated sevoflurane-induced iron overload and lipid peroxide accumulation. Our study elucidated that the miR-182-5p/GPX4 pathway was implicated in sevoflurane-induced ototoxicity by promoting ferroptosis.

## 1. Introduction

Sevoflurane is a commonly used inhalational general anesthetic in clinical practice. It is widely used in pediatric surgery and outpatient surgery because of its advantages of rapid induction, low respiratory irritation, mild circulatory inhibition, and rapid awakening [1]. However, the effects of sevoflurane on the developing nervous system have raised concerns within the medical community [2]. Several studies have reported that sevoflurane can induce central nervous system toxicity, leading to neuronal death through mechanisms such as the accumulation of reactive oxygen species (ROS), mitochondrial dysfunction, the release of pro-inflammatory factors, and the polarization of microglial cells towards M1, ultimately resulting in cognitive dysfunction [3,4,5,6]. Similarly, the auditory nervous system can be affected by environmental factors and drugs, potentially leading to hearing loss [7,8]. Our previous study [9] demonstrated that sevoflurane affects the normal function of hair cells by promoting ROS accumulation in the hair cells of neonatal mice, which subsequently contributes to the development of hearing loss in adult mice.

Ferroptosis is a new form of cell death brought on by intracellular Fe^2+^ overload, which results in an excess of toxic lipid peroxides. The key mechanism of ferroptosis involves the depletion of glutathione, which leads to a decrease in the activity of Glutathione Peroxidase 4 (GPX4). This enzyme is responsible for metabolizing lipid peroxides, and its decreased activity allows for the oxidation of lipids by Fe^2+^ to produce reactive oxygen species [10]. Ferroptosis has been implicated in various pathological processes, including cardiovascular disease, inflammation, neurodegenerative diseases, and cancer [11,12,13,14]. In the context of hearing damage, ferroptosis has been linked to the ototoxic effects of cisplatin, which induce ferroptosis in the inner ear, along with the excessive generation of reactive oxygen species, the activation of apoptosis, and the accumulation of intracellular lipid peroxidation [15,16]. Similarly, neomycin has been shown to induce intracellular Fe^2+^ accumulation and lipid peroxidation in the HEI-OC1 cell line and cochlear hair cells [17]. Studies have also demonstrated that indicators of ferroptosis, such as Fe^2+^, malondialdehyde (MDA), and lactotransferrin (LTF), were elevated in aged HEI-OC1 cells, cochlear explants, and cochlear tissue [18]. While studies have shown that ferroptosis-related genes play a critical role in hippocampal neuronal damage associated with sevoflurane exposure [19,20,21,22], there is currently no research on whether ferroptosis is involved in sevoflurane-induced ototoxicity.

MicroRNAs (miRNAs) are short (approximately 23 nucleotides) non-coding RNA strands that downregulate the expression of target genes by binding to the 3′UTR of the target gene’s mRNA. miRNAs need to bind to the mRNAs of their target genes in order to fulfill their biological roles in cellular differentiation, apoptosis and proliferation, autophagy, and immune response [23]. In the auditory system, miRNAs are involved in the development of the auditory system and are implicated in hearing loss. For example, miR-106a promoted oxidative stress in a rat model of gentamicin-induced deafness by targeting connexin-43 [24]. Additionally, the overexpression of lncRNAH19 could inhibit oxidative stress-induced hair cell apoptosis and ameliorate hearing loss in age-related hearing loss (ARHL) by regulating miR-653-5p, which in turn regulates SIRT1 expression [25]. Furthermore, miR-96 and miR-183 played crucial roles in synaptic transmission in the auditory brainstem [26]. MiRNAs also play a regulatory role in ferroptosis, and studies have shown that the miRNA/GPX4 pathway regulates GPX4 expression in cells undergoing ferroptosis in various diseases [27]. Additionally, miRNAs have been implicated in sevoflurane-induced ferroptosis-related neurotoxicity [20]. However, there is limited research on whether the miRNA/GPX4 pathway is involved in ferroptosis in the inner ear. Our study aims to investigate whether ferroptosis contributes to sevoflurane-induced ototoxicity in inner ear hair cells and whether the miRNA/GPX4 pathway is involved in this process.

## 2. Results

### 2.1. Fer-1 Attenuated the Ototoxic Effects of Sevoflurane in Mice

**Figure 1:**
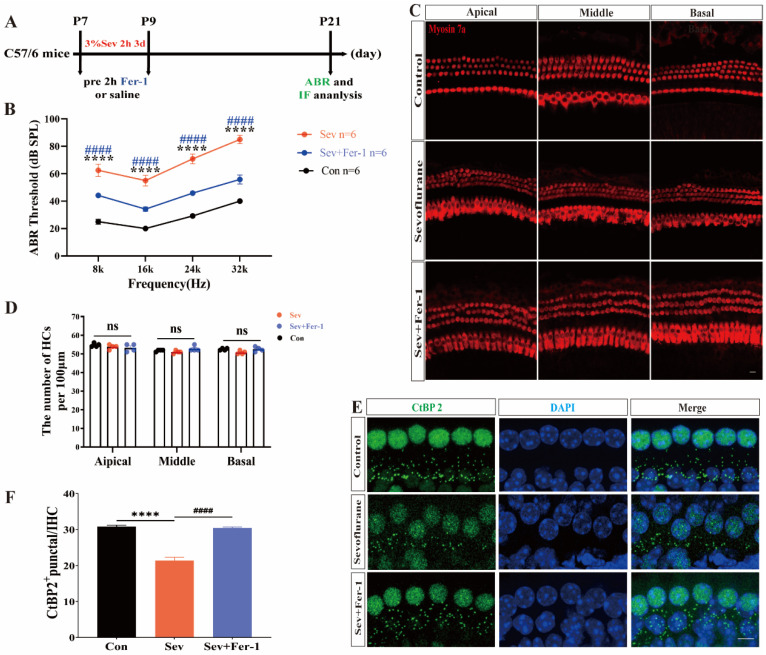
Fer-1 attenuated the ototoxic effect of sevoflurane in mice. (**A**) Schematic of the experimental procedure: P7 C57/6 mice received 20 mg/kg of Fer-1 2 h before each 3-day sevoflurane anesthesia session at 3% concentration for 2 h. At P21, mice underwent ABR testing, followed by cochlea dissection for immunofluorescence staining. (**B**) Auditory brainstem response (ABR) thresholds in sevoflurane-exposed mice were significantly elevated at 8 kHz, 16 kHz, 24 kHz, and 32 kHz compared to controls, with decreased thresholds after Fer-1 pretreatment. Each group included 6 mice. (**C**) Immunofluorescence staining of myosin 7a (red) in the apical, middle, and basal cochlear rings showed no statistical difference in hair cell counts among the three groups; scale bar = 20 μm. (**D**) Hair cell count. n = 4 cochleae per group. (**E**) Immunofluorescence staining for CtBP2 (green) and DAPI (blue) in the middle cochlear ring showed significant differences among the three groups; scale bar = 20 μm. (**F**) CtBP2 counts. n = 4 cochleae per group. Data were expressed as mean ± SEM. **** *p* < 0.0001 vs. Con; ^####^ *p* < 0.0001 vs. Sev + Fer-1; ns indicates that the difference was not statistically significant.

To investigate the potential role of ferroptosis in sevoflurane-induced hearing damage in mice, we followed the experimental procedure outlined in Figure 1A. We assessed the hearing thresholds of the mice using the auditory brainstem response (ABR) test, as illustrated in Figure 1B. Compared to the control group, the hearing thresholds of mice anesthetized with sevoflurane were significantly elevated at 8 kHz, 16 kHz, 24 kHz, and 32 kHz, indicating a decrease in the hearing function of the sevoflurane-exposed mice. However, pretreatment with the ferroptosis inhibitor ferrostatin-1 (Fer-1) resulted in decreased hearing thresholds and the recovery of hearing function in sevoflurane-exposed mice (Figure 1B). Following the completion of the ABR test, we dissected the cochleae of mice to investigate the potential cause of sevoflurane-induced hearing loss and performed immunofluorescence staining. Surviving hair cells were labeled using myosin 7a (a hair cell marker), and ribbon synapses of hair cells were labeled with CtBP2 (a presynaptic protein marker). The number and morphology of hair cells in sevoflurane-exposed mice were not significantly different from those in the control group (Figure 1C,D). However, Figure 1E, F show a significant reduction in the number of CtBP2-positive puncta in the cochleae of sevoflurane-exposed mice compared to the control group, suggesting a significant loss of cochlear synapses in sevoflurane-exposed mice. As anticipated, the number of CtBP2-positive puncta increased following pretreatment with Fer-1, indicating a decrease in ribbon synapse loss (Figure 1E,F).

These results suggested that ferroptosis was involved in sevoflurane-induced hearing damage in mice and that the inhibition of ferroptosis can rescue the ototoxic effects of sevoflurane in mice.

### 2.2. Fer-1 Attenuated Sevoflurane-Induced Ototoxic Effects in Cochlear Explants

**Figure 2:**
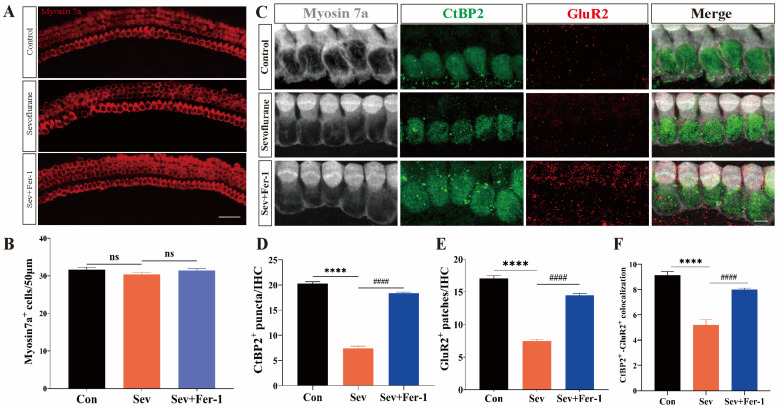
Fer-1 attenuated sevoflurane-induced ototoxic effects in cochlear explants. (**A**) Immunofluorescence staining of myosin 7a (red) in hair cells in the middle turn of cochlear explants from the three groups; scale bar = 50 μm. (**B**) Hair cell counts. n = 5 cochleae per group. (**C**) Immunofluorescence staining for myosin 7a (gray), CtBP2 (green) and GluR2 (red) in the middle ring of cochlear explants from the three groups; scale bar = 10 μm. (**D**) CtBP2 counts. n = 9 cochleae per group. (**E**) GluR2 counts. n = 9 cochleae per group. (**F**) CTBP2 and GluR2 colocalization counts. n = 9 cochleae per group. Data were presented as mean ± SEM. **** *p* < 0.0001 vs. Con; ^####^ *p* < 0.0001 vs. Sev + Fer-1; ns indicates that the difference was not statistically significant.

Following the confirmation of the ototoxicity of sevoflurane in vivo, we proceeded to validate its effects on cochlear explants. Immunofluorescence staining was performed on the treated cochlear explants using myosin 7a, CtBP2, and GluR2 to label surviving hair cells and ribbon synapses, respectively. Consistent with the in vivo results, there were no differences in the number and morphology of hair cells among the sevoflurane-exposed group, the Fer-1 pretreatment group, and the control group (Figure 2A,B). However, the number of CtBP2-positive puncta, GluR2-positive puncta, and CtBP2/GluA2 double-positive puncta in cochlear explants from the sevoflurane-exposed group was significantly reduced compared to the control group. Pretreatment with Fer-1 mitigated the sevoflurane-induced reduction in the number of CtBP2-positive puncta, GluR2-positive puncta, and CtBP2/GluA2 double-positive puncta in cochlear explants (Figure 2C–F).

These findings suggested that the ototoxicity of sevoflurane, both in vivo and in vitro, primarily manifests as damage to the ribbon synapses, without significantly affecting the number and morphology of hair cells.

### 2.3. Sevoflurane-Induced Ferroptosis in Cochlear Explants

To investigate whether ferroptosis was involved in sevoflurane exposure-induced hearing damage, we used FO fluorescent probes and BODIPY fluorescent probes to detect iron overload and lipid peroxide accumulation in cochlear explants. Red fluorescent signals represent the reduced state, while green fluorescent signals represent the oxidized state.

**Figure 3:**
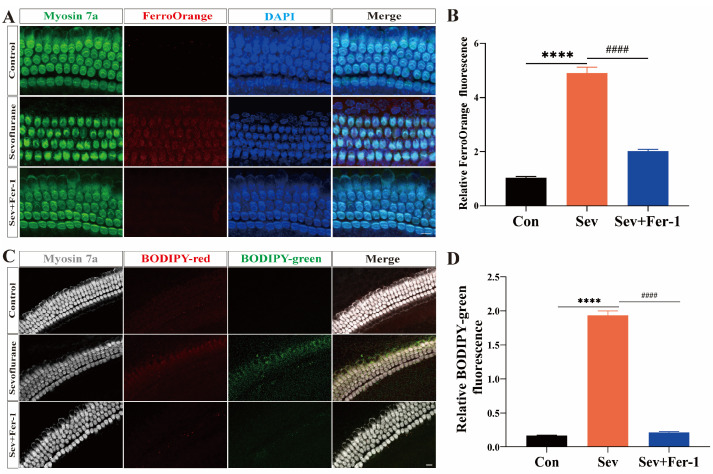
Sevoflurane-induced ferroptosis in cochlear explants. (**A**) FerroOrange red fluorescent probe detected Fe^2+^ accumulation, and immunofluorescence staining of myosin 7a (green) and DAPI (blue) in the cochlear explants in three groups; scale bar = 10 μm. n = 6 cochleae per group. (**B**) were quantitatively analyzed for (**A**). (**C**) BODIP 581/591 C11 (red and green) assay for lipid peroxide accumulation in the cytoplasm and immunofluorescence staining of myosin 7a (gray) of the three cochlear explants groups; scale bar = 10 μm. n = 6 cochleae per group. (**D**) Quantification of (**C**) using ImageJ 2.3.0/1.53S. Data were expressed as mean ± SEM. **** *p* < 0.0001 vs. Con; ^####^ *p* < 0.0001 vs. Sev + Fer-1.

As shown in Figure 3A,B, in cochlear explants from the sevoflurane-exposed group, the red fluorescence intensity of FerroOrange was significantly enhanced compared with the control group. However, pretreatment with Fer-1 attenuated the red fluorescence intensity of FerroOrange. These results suggested that sevoflurane exposure led to iron overloading in cochlear explants, and Fer-1 could mitigate this effect. Using BODIP 581/591 C11 immunofluorescence staining to detect lipid peroxidation, this study found that the relative green fluorescence intensity was significantly increased in the sevoflurane-exposed group compared with the control group, suggesting that more lipids were oxidized in the cochlear explants of the sevoflurane-exposed group, leading to lipid peroxide accumulation (Figure 3C,D). However, pretreatment with Fer-1 significantly reduced the relative green fluorescence intensity in sevoflurane-exposed cochlear explants, indicating less oxidation of lipids and reduced lipid peroxide accumulation (Figure 3C,D).

The findings indicated that sevoflurane exposure induced ferroptosis in cochlear explants, while Fer-1 can alleviate this effect.

### 2.4. Sevoflurane-Induced Ferroptosis in HEI-OC1 Cells

**Figure 4:**
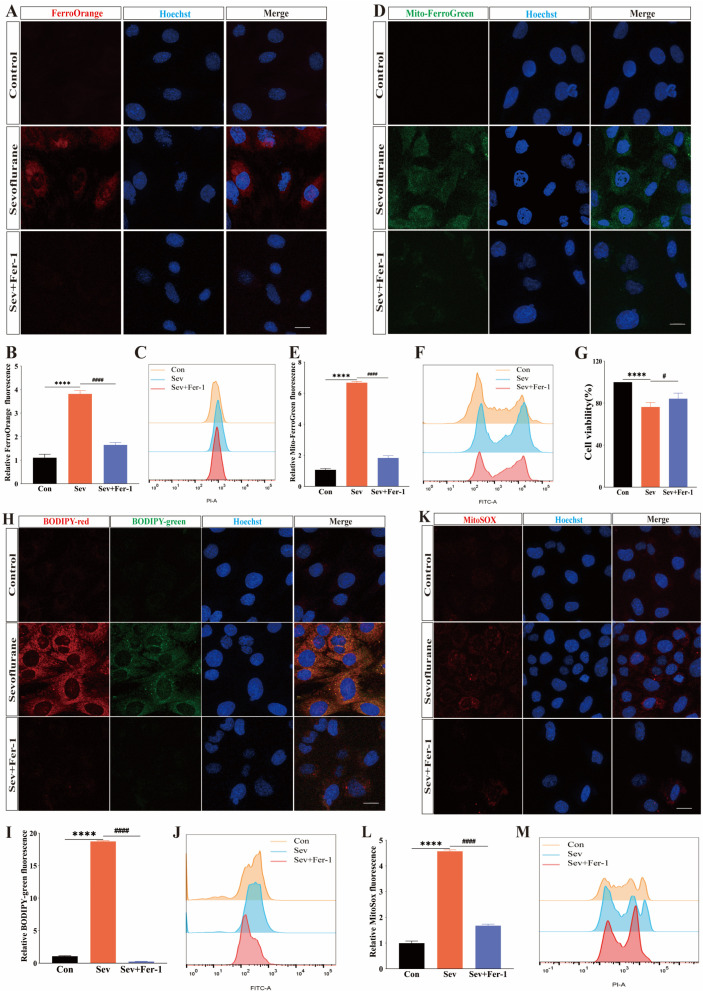
Sevoflurane-induced ferroptosis in HEI-OC1 cells. (**A**) FerroOrange red fluorescent probe detected Fe^2+^ accumulation in the cell cytoplasm, and immunofluorescence staining of hoechst (blue) in three groups; scale bar = 10 μm. (**B**,**C**) were quantitatively analyzed for (**A**) using ImageJ and flow cytometry experiments, respectively. (**D**) Mito-FerroGreen green fluorescent probe detected Fe^2+^ accumulation in mitochondria, and immunofluorescence staining of hoechst (blue) in three groups; scale bar = 10 μm. (**E**,**F**) were quantitatively analyzed for (**D**) using ImageJ and flow cytometry experiments, respectively. (**G**) Cell viability was tested for all three groups of cells. Each experiment was repeated at least three times. (**H**) BODIP 581/591 C11 assay for lipid peroxide accumulation in the cytoplasm of the three cell groups; scale bar = 10 μm. (**I**) Quantification of (**H**) using ImageJ. (**J**) Quantification of (**H**) using flow cytometry. (**K**) MitoSOX assay for lipid peroxide accumulation in the mitochondria of the three groups; scale bar = 10 μm. (**L**) Quantification of (**K**) using ImageJ. (**M**) Quantification of (**K**) using flow cytometry. Each experiment was repeated at least three times. Data were expressed as mean ± SEM. **** *p* < 0.0001 vs. Con; ^#^ *p* < 0.05, ^####^ *p* < 0.0001 vs. Sev + Fer-1.

To investigate the mechanism underlying sevoflurane-induced ototoxicity, HEI-OC1 cells were selected as the experimental model. Initially, cell viability experiments were conducted using the CCK-8 kit, revealing that sevoflurane exposure decreased the cell viability of HEI-OC1 cells compared to the control group. However, pretreatment with Fer-1 rescued the decrease in cell viability caused by sevoflurane (Figure 4G).

Next, to assess whether sevoflurane induced iron overload in HEI-OC1 cells, the FerroOrange red fluorescent probe and Mito-FerroGreen green fluorescent probe were employed to detect Fe^2+^ accumulation in cells and mitochondria, respectively. Flow cytometry experiments revealed significantly higher FerroOrange red fluorescence signals (Figure 4A–C) and Mito-FerroGreen green fluorescence signals (Figure 4D–F) in the HEI-OC1 cells of the sevoflurane-exposed group compared to the control group, indicating increased Fe^2+^ accumulation in the cells and mitochondria, indicative of iron overload. Notably, pretreatment with Fer-1 reduced the elevated FerroOrange red fluorescence signal (Figure 4A–C) and Mito-FerroGreen green fluorescence signal (Figure 4D–F) in HEI-OC1 cells after sevoflurane exposure, thereby inhibiting sevoflurane-induced iron overload. These results suggested that sevoflurane exposure leads to iron overload in the cytoplasm and mitochondria of HEI-OC1 cells, which could be mitigated by pretreatment with Fer-1. 

Iron overload could lead to the accumulation of lipid peroxides in cells. To label lipid peroxides in HEI-OC1 cells and mitochondria, BODIP 581/591 C11 fluorescent dye (red fluorescent signals represent the reduced state and green fluorescent signals represent the oxidized state) and a MitoSOX red fluorescent probe were used, respectively. The BODIP 581/591 C11 immunofluorescence staining and flow cytometry experiments revealed a significant increase in the green fluorescent signal in the sevoflurane-exposed group compared to the control group (Figure 4H–J). However, after pretreatment with Fer-1, the green fluorescence signal was reduced in HEI-OC1, indicating a decrease in lipid peroxide production (Figure 4H–J). Using a MitoSOX red fluorescent probe, we labeled lipid peroxides in HEI-OC1 mitochondria. The red fluorescent signal was higher in HEI-OC1 cells exposed to sevoflurane than in the control group (Figure 4K–M). This suggested that more lipid peroxides were produced in the mitochondria of cells in the sevoflurane-exposed group. As anticipated, Fer-1 pretreatment weakened the red fluorescence signal in HEI-OC1 cells (Figure 4K–M), indicating a reduction in mitochondrial lipid peroxide production.

Collectively, these experimental results suggested that sevoflurane impaired the antioxidant capacity of HEI-OC1 cells and triggered ferroptosis.

### 2.5. MiR-182-5p Targeted GPX4 in HEI-OC1 Cells

Having established the involvement of ferroptosis in sevoflurane-induced ototoxicity in HEI-OC1 cells, the next step was to investigate the mechanism by which sevoflurane induced ferroptosis in HEI-OC1 cells.

**Figure 5:**
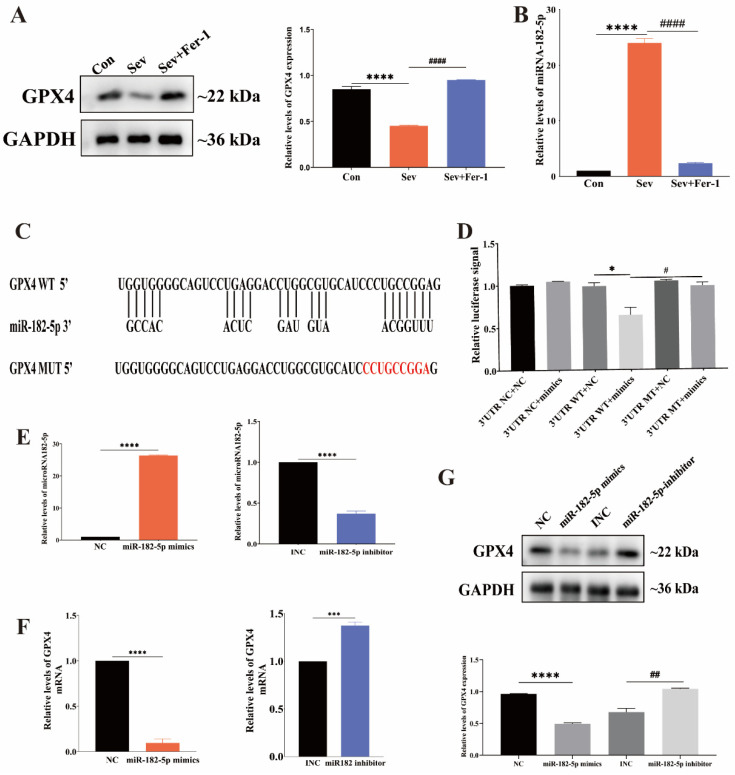
MiR-182-5p targeted GPX4 in HEI-OC1 cells. (**A**) Western blot analysis was conducted to measure the relative expression of the GPX4 protein. (**B**) qRT-PCR was used to measure the relative expression of miR-182-5p. (**C**) Predicted binding site of miR-182-5p on the GPX4 mRNA 3′UTR. (**D**) Luciferase activity was determined in different experimental groups. (**E**) qRT-PCR was performed to measure the relative expression of miR-182-5p mimics and inhibitors. (**F**) qRT-PCR was conducted to detect the relative expression of GPX4 mRNA in cells treated with miR-182-5p mimics and inhibitors. (**G**) Western blot was performed to measure the relative expression of the GPX4 protein. Each experiment was repeated at least three times. Data were expressed as mean ± SEM. * *p* < 0.05, *** *p* < 0.001, **** *p* < 0.0001 vs. Con; ^#^ *p* < 0.05, ^##^ *p* < 0.01, ^####^ *p* < 0.0001 vs. Sev + Fer-1.

GPX4 is a major regulator of ferroptosis and is known to play a crucial role in counteracting it. To determine whether sevoflurane treatment affected GPX4 expression in HEI-OC1 cells, we conducted a Western blot analysis. Figure 5A showed that GPX4 expression was significantly lower in the sevoflurane-exposed group than in the control group, and Fer-1 pretreatment increased GPX4 expression in HEI-OC1 cells, as indicated by the relative gray values of the bands from the Western blot.

MicroRNAs (miRNAs) have been shown to play a significant role in controlling GPX4 expression in cells undergoing ferroptosis. In our study, we focused on microRNA-182-5p (miR-182-5p), which has been implicated in ferroptosis regulation in other studies. We used qRT-PCR to determine if miR-182-5p expression changed in HEI-OC1 cells following sevoflurane exposure. The results showed that the expression level of miR-182-5p was significantly higher in HEI-OC1 cells from the sevoflurane-exposed group compared to the control group, while the expression level of miR-182-5p was significantly lower in HEI-OC1 cells treated with Fer-1 (Figure 5B).

For the regulation of the target gene, the microRNA must attach to the 3′UTR end of its mRNA. To investigate whether the 3′UTR end of GPX4 mRNA can bind to miRNA-182-5p, we used the bioinformatics website Bielefeld Bioinformatics Service (https://bibiserv.cebitec.uni-bielefeld.de/ (accessed on 20 November 2023)). to make predictions, which revealed that the two have a binding site (Figure 5C). Additionally, we performed dual luciferase reporter gene analysis for more precise verification. GPX4 3′UTR WT plasmid-transfected cells’ luciferase activity was considerably suppressed by miR-182-5p mimics but not GPX4 3′UTR MUT-transfected cells (Figure 5D). This suggested that miR-182-5p could bind to GPX4 mRNA at the 3′UTR end of the mRNA and that the two could interact.

Subsequently, HEI-OC1 cells were treated with miR-182-5p mimics and inhibitors to investigate the role of miR-182-5p in regulating ferroptosis by targeting GPX4. Figure 5E showed that mimics and inhibitors successfully modulated miR-182-5p expression in HEI-OC1 cells. Western blot and qRT-PCR confirmed that the upregulation of miR-182-5p decreased GPX4 expression at both the mRNA and protein levels, while the downregulation of miR-182-5p increased GPX4 expression (Figure 5F,G).

In summary, miR-182-5p negatively regulated GPX4 expression by directly binding to the 3′UTR end of GPX4 mRNA in HEI-OC1 cells undergoing ferroptosis induced by sevoflurane.

### 2.6. Inhibition of miR-182-5p Attenuated Sevoflurane-Induced Ferroptosis in HEI-OC1 Cells

We transfected miR-182-5p inhibitors into HEI-OC1 cells before sevoflurane treatment to investigate whether this approach could mitigate sevoflurane-induced ferroptosis in these cells.

**Figure 6:**
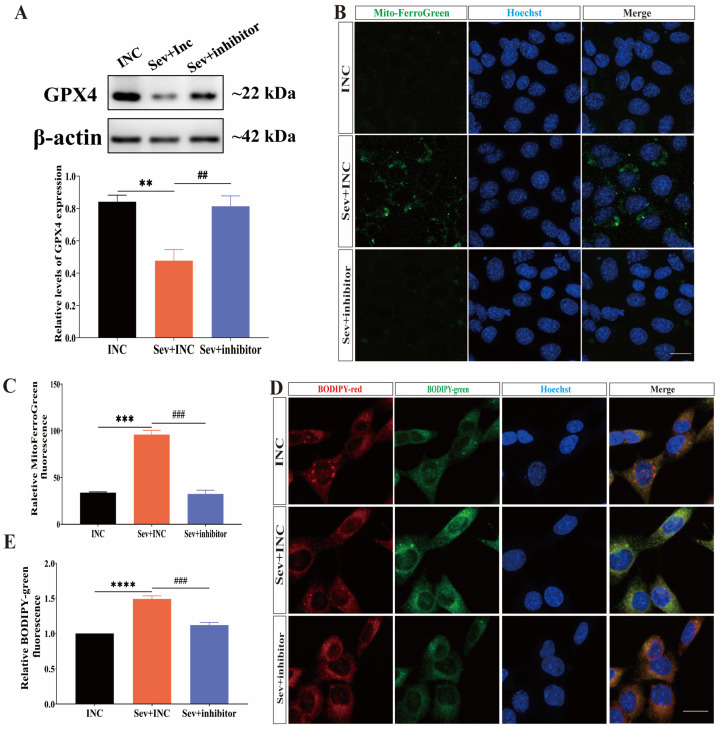
Inhibition of miR-182-5p attenuated sevoflurane-induced ferroptosis in HEI-OC1 cells. (**A**) Western blot analysis was conducted to measure GPX4 protein expression levels. (**B**) Mito-FerroGreen green fluorescent probe was used to visualize Fe^2+^ accumulation in mitochondria, and immunofluorescence staining of hoechst (blue) in three groups; scale bar = 10 μm. (**C**) Quantitative analysis of (**B**) was performed. (**D**) BODIP 581/591 C11 (red and green) assay for lipid peroxide accumulation in the cytoplasm, and immunofluorescence staining of hoechst (blue) of the three cell groups; scale bar = 10 μm. (**E**) Quantitative analysis of (**D**) was conducted. Each experiment was repeated at least three times. Data were expressed as mean ± SEM. ** *p* < 0.01, *** *p* < 0.001, **** *p* < 0.0001 vs. Con; ^##^ *p* < 0.01, ^###^ *p* < 0.001 vs. Sev + Fer-1.

Western blot analysis (Figure 6A) showed that transfecting HEI-OC1 cells with miR-182-5p inhibitors before sevoflurane exposure significantly increased the expression level of GPX4 compared to the group exposed to sevoflurane alone. This indicated that the inhibition of miR-182-5p could reverse the decrease in GPX4 expression induced by sevoflurane exposure in HEI-OC1 cells.

To assess whether miR-182-5p inhibitors could alleviate iron overload in HEI-OC1 cells following sevoflurane exposure, we performed Mito-FerroGreen immunofluorescence staining. The intensity of green fluorescence signals was significantly reduced in HEI-OC1 cells transfected with miR-182-5p inhibitors before exposure to sevoflurane compared to the group exposed to sevoflurane alone (Figure 6B,C), indicating a decrease in the content of Fe^2+^ in the mitochondria. Furthermore, to determine whether miR-182-5p inhibitors could reverse the accumulation of lipid peroxides in HEI-OC1 cells after exposure to sevoflurane, we used BODIP 581/591 C11 immunofluorescence staining to detect lipid peroxides in the cells. The intensity of green fluorescent signals was significantly reduced in HEI-OC1 cells transfected with miR-182-5p inhibitors before anesthetic exposure (Figure 6D,E), suggesting a reduction in lipid peroxidation in the cells.

In conclusion, our findings demonstrated that the inhibition of miR-182-5p attenuated sevoflurane-induced ferroptosis in HEI-OC1 cells by targeting GPX4.

## 3. Discussion

As a commonly used general inhalational anesthetic in clinical practice, sevoflurane’s neurotoxicity has been a major concern in the medical community. According to earlier research, sevoflurane caused M1 polarization in microglia [28], increased the production of inflammatory cytokines [4], made mitochondrial autophagy abnormal and dysfunctional [29], prevented the growth of developing neurons [29], and eventually caused the development of developmental neurons to undergo apoptosis. These effects ultimately lead to apoptosis of developmental neuronal cells and cognitive dysfunction. Zhao et al. demonstrated that sevoflurane caused developmental neurotoxicity through 15LO2-mediated iron overload [20]. Feng et al. showed that endoplasmic reticulum stress-mediated activation of ATF3 contributes to sevoflurane-induced neuronal iron overload via H_2_O_2_ accumulation and resulting iron overload [30]. Another study showed that sevoflurane caused mitochondrial ROS production and elevated cytoplasmic calcium levels, triggering the opening of the mitochondrial permeability transition pore (MPTP) and lowering the mitochondrial membrane potential (MMP), inducing mitochondrial iron overload, and ultimately leading to cognitive dysfunction [31]. The peripheral auditory nervous system is similarly susceptible to damage by drugs and the environment [7,32,33]. Our previous study showed that sevoflurane was capable of producing ototoxicity in the developing peripheral auditory system by inducing oxidative stress [9]. In this study, we investigated whether ferroptosis was involved in sevoflurane-induced ototoxicity and attempted to explore the underlying mechanisms. 

Lipid peroxidation is a key feature of ferroptosis and can lead to extensive cellular damage. In our previous study, we observed that sevoflurane did not affect the number or morphology of hair cells. However, sevoflurane attenuated the triggered vesicle release from inner hair cell synaptic ribbons [9] In this study, we found that sevoflurane exposure specifically affected the ribbon synapses, confirming that synaptic structures are particularly vulnerable to oxidative damage induced by sevoflurane. This susceptibility may result in structural and functional disruptions of the synapses before other cellular components are affected. This finding suggests a specific vulnerability of ribbon synapses to oxidative stress, which may explain the observed functional impairment. 

This study utilized both in vitro and in vivo experiments to elucidate the contribution of ferroptosis to sevoflurane-induced ototoxicity. P7 mice were administered Fer-1 before anesthesia. The auditory brainstem response (ABR) thresholds of mice in the ferrostatin-1-pretreated group were significantly lower than those in the group anesthetized with sevoflurane alone, indicating the involvement of ferroptosis in sevoflurane-induced hearing loss in mice. Using FerroOrange and Mito-FerroGreen fluorescent probes to detect intracellular and mitochondrial divalent iron, respectively, considerable iron overload was observed in both the cytosol and mitochondria of HEI-OC1 cells following sevoflurane exposure. Additionally, the accumulation of lipid peroxides in the cytoplasm and mitochondria of the cells was detected using BODIP 581/591 C11 and MitoSOX, respectively. Significant accumulation of lipid peroxides was observed in both the cytoplasm and mitochondria of HEI-OC1 cells after sevoflurane treatment. Importantly, these phenomena were reversed by the ferroptosis inhibitor, confirming the involvement of ferroptosis in the ototoxic effect of sevoflurane in HEI-OC1 cells.

GPX4 functions as a central regulatory enzyme in ferroptosis, playing a crucial role in its onset. By catalyzing the conversion of glutathione to oxidized glutathione, GPX4 can transform harmful lipid peroxides into harmless cellular byproducts, thereby mitigating the accumulation of excessive lipid peroxides within cells [15,34,35]. The inhibition of GPX4 led to the accumulation of lipid peroxides, resulting in ferroptosis [36]. Several studies have shown that GPX4 expression was negatively regulated by various microRNAs. For example, the miR-134-5p/GPX4 axis regulated iron overload in bronchopulmonary dysplasia (BPD) [37]. Additionally, the SMAD3/miR-129-2-3p/GPX4 axis mediated macrophage-induced iron overload in sepsis [38], and the miR-125b-5p/GPX4 axis was involved in iron overload in acute lung injury in sepsis [39]. It has also been demonstrated that miR-182-5p regulated ferroptosis in ischemia/reperfusion (I/R)-induced kidney injury [40]. We performed bioinformatics analysis and identified GPX4 as a potential target gene of miR-182-5p.

**Figure 7:**
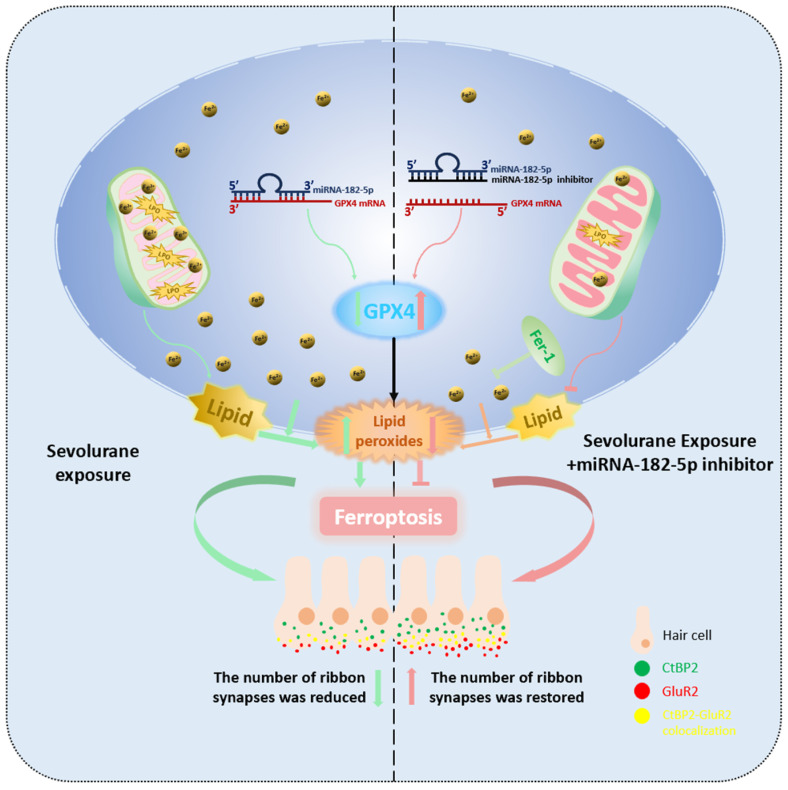
The miR-182-5p/GPX4 pathway contributes to sevoflurane-induced ototoxicity via ferroptosis.

The novelty of our study lay in demonstrating that ferroptosis was responsible for sevoflurane-induced ototoxicity and elucidating the possible mechanisms. As shown in Figure 7, we found that the expression level of miR-182-5p increased in hair cells after sevoflurane exposure, and miR-182-5p could directly target the 3′UTR of GPX4 mRNA involved in ferroptosis. The inhibition of miR-182-5p expression attenuated sevoflurane-induced ferroptosis-related ototoxicity. Our study complemented previous studies on the neurotoxicity of sevoflurane to the peripheral auditory system and provided new ideas and targets for the prevention and treatment of sevoflurane-induced ototoxicity.

However, our study has limitations. Firstly, while sevoflurane treatment altered the activity of HEI-OC1 cells, it had no effect on the quantity and shape of hair cells in vivo or in cochlear explants. This may be because HEI-OC1 cells lack the protective influence of support cells [41,42,43] or because the effects of sevoflurane on these cells were not sufficient to cause cell death when balanced against intracellular protective factors [44,45]. Secondly, ferroptosis is a complex process [46], and our study only focused on GPX4. Other regulators of ferroptosis, such as SLC7A11, ACSL4, and ferritin, may also be involved in sevoflurane-induced ototoxicity, which requires further investigation.

## 4. Materials and Methods

### 4.1. Animals and Anesthesia Treatment

We performed in vivo experiments on C57BL/6 mice at 7 days postnatal (P7) age, randomly assigning them to three groups: the Con group, the Sev group, and the Sev + Fer-1 group. Mice in the sevoflurane-exposed group received 3% sevoflurane anesthesia for 2 h daily for three consecutive days (P7–P9) in a closed chamber containing 3% sevoflurane carried by 60% oxygen and 40% nitrogen. Throughout anesthesia, a heating device maintained the mice’s rectal temperature at 37 ± 0.5 °C to ensure stable vital signs. Sevoflurane concentration was monitored using a Vamos side-stream gas analyzer (Dräger, Lübeck, Germany) to maintain a steady 3% concentration. Control mice were kept in a closed chamber with 60% oxygen and 40% nitrogen under identical conditions. Ferrostatin-1-pretreated mice received 20 mg/kg of Fer-1(MedChemExpress, Shanghai, China) via intraperitoneal injection 2 h before each anesthesia session, with all other conditions matching those of the sevoflurane-exposed group. All animals were housed in a quiet environment at 21–23 °C with a controlled 12 h light–dark cycle and had ad libitum access to water and food. The animal experiments and research protocols were approved and conducted in accordance with the Institutional Animal Care and Use Committee of Fudan University.

### 4.2. Cochlear Explant Culture and Treatment

The cochleae of C57BL/6 mice at P2 were dissected in ice-cold PBS, and the basement membrane was carefully removed. The basilar membranes of the ear were then attached to a circular slide coated with Cell-Tak (BD Biosciences, Franklin Lakes, NJ, USA). Cochlear explants were cultured in DMEM/F12 medium (Hyclone, Logan, UT, USA) supplemented with 1% N-2 Supplement, 1% B-27 Serum-Free Supplement (Invitrogen, Carlsbad, CA, USA), and 50 IU/mL ampicillin, and incubated in a 37 °C incubator with 5% CO_2_/95% air.

The cochlear explants were randomly divided into three groups: the Con group, the Sev group, and the Sev + Fer-1 group. Cochlear explants in the Sev group were treated with 3% sevoflurane for 6 h in a closed chamber. Cochlear explants in the Sev + Fer-1 group were pretreated with 40 μM ferrostatin-1 added to the culture medium 2 h before sevoflurane exposure. The cochlear explants in the Con group were incubated at 37 °C in a closed chamber with 5% CO_2_ and 92% air.

### 4.3. HEI-OC1 Cell Culture and Treatment

HEI-OC1 cells were cultured in high-glucose DMEM (Gibco, Thermo Fisher Scientific, Waltham, MA, USA) supplemented with 10% Fetal Bovine Serum (FBS) (Gibco, Thermo Fisher Scientific, USA) and maintained in an incubator at 37 °C with 5% CO_2_. Cells were passaged using trypsin (Gibco, Thermo Fisher Scientific, USA) when the cell density reached 80%.

HEI-OC1 cells were randomly divided into three groups: the Con group, the Sev group, and the Sev + Fer-1 group. Cells in the Sev group were cultured continuously for 6 h at 37 °C with 5% CO_2_ in a closed chamber containing 3% sevoflurane. Two hours before the start of anesthesia, 40 μM Fer-1 was added to the cell culture medium of the Sev + Fer-1 group. Cells in the Con group were cultured under the same conditions in a closed chamber without sevoflurane gas at 37 °C with 5% CO_2_.

### 4.4. Acquisition of Mouse Cochlear Basement Membrane

The mice were euthanized by cervical dislocation, and the cochleae were quickly dissected into ice-cold phosphate-buffered saline (PBS). A 1 mL syringe needle was used to perforate the top of the cochleae, and 4% paraformaldehyde (PFA) was injected to flush out any remaining blood. The cochleae were then fixed in 4% PFA for 2 h at room temperature on a shaker, followed by three washes with PBS for 10 min each. Decalcification was performed using a 10% EDTA solution for 3 h at room temperature on a shaker. The cochleae were placed in ice-cold PBS, and the cochlear basement membrane was processed under a microscope to isolate the three rings.

### 4.5. Immunofluorescence Staining

After fixation in 4% PFA for 30 min, the cochleae were washed three times with 1% PBS. Subsequently, the cochleae were permeabilized with 1% Triton X-100 in PBS (PBST) for 30 min and then blocked with PBST containing 10% donkey serum for 1 h. The cochleae were then incubated overnight at 4 °C with the following primary antibodies: anti-myosin 7a antibody (1:500; BD Biosciences, USA), anti-glutamate receptor 2, extracellular, clone 6C4 (GluR2; 1:200; Millipore, Schwalbach, Germany), and anti-CtBP2 antibody (1:500; BD Biosciences, USA). After washing the samples three times with 1% PBS, they were incubated overnight at 4 °C with the corresponding secondary antibody. Following three washes with 1% PBS, cochleae were stained with 4′6-diamidino-2-phenylindole (DAPI; Beyotime, Shanghai, China) for 10 min at room temperature. Finally, the cochleae were flattened on a clean slide, drops of Antifade Mounting Medium (Beyotime, Shanghai, China) were added, and then they were covered with a coverslip and sealed with nail polish. The samples were observed under a confocal fluorescence microscope (Leica SP8; Leica Microsystems, Wetzlar, Germany).

### 4.6. Cell Viability Test

Cell viability was assessed using the Cell Counting Kit-8 (CCK-8, Sigma, Steinheim, Germany). HEI-OC1 cells were seeded at 5000 cells per well in 96-well plates and cultured at 37 °C with 5% CO_2_. After treatment, 200 μL of medium containing 10% CCK-8 solution was added to each well. The absorbance at 450 nm was measured using a microplate reader (Bio-Rad, Hercules, CA, USA) after incubation at 37 °C with 5% CO_2_ for 2 h.

### 4.7. Fe^2+^ Measurement

To assess iron overload in sevoflurane-treated cochlear explants and cells, we used FerroOrange and Mito-FerroGreen fluorescent probes (DOJINDO, Kumamoto, Japan) to detect intracellular and intramitochondrial divalent iron ions, respectively. After treatment, HEI-OC1 cells were incubated in fresh media overnight to recover. Subsequently, cells were incubated with FerroOrange and Mito-FerroGreen (1:1000 dilution) for 30 min at 37 °C with 5% CO_2_; cochlear explants were incubated with FerroOrange (1:1000 dilution) for 30 min at 37 °C with 5% CO_2_. After washing the cells, they were stained with the cytosolic fluorescent marker Hoechst (1:150 dilution; DOJINDO, Japan) for 10 min at 37 °C with 5% CO_2_. Cochlear explants were stained with anti-myosin 7a antibody (1:500; BD Biosciences, USA). After washing the samples three times with 1% PBS, they were incubated overnight at 4 °C with the corresponding secondary antibody. Then, cochlear explants were stained with the cytosolic fluorescent marker DAPI (Beyotime, Shanghai, China) for 10 min at room temperature. The samples were then examined using a Leica SP8 confocal fluorescence microscope (Leica SP8; Leica Microsystems, Wetzlar, Germany). Flow cytometry was used to measure fluorescence intensity, and FlowJo v10 was used to analyze relative fluorescence intensity.

### 4.8. ROS Measurement

To assess the production of excessive lipid peroxides in cochlear explants and HEI-OC1 cells after sevoflurane treatment, we examined the accumulation of lipid peroxides in cells and mitochondria using BODIP 581/591 C11 (Invitrogen, USA) and MitoSOX (Invitrogen, USA), respectively. HEI-OC1 cells were treated and then co-incubated with BODIP 581/591 C11 (1:1000 dilution) and MitoSOX (1:1000 dilution) for 30 min. Subsequently, cells were washed with PBS and incubated with Hoechst (1:150 dilution) for 10 min at 37 °C with 5% CO_2_. Cochlear explants were incubated with BODIP 581/591 C11 (Invitrogen, USA) (1:1000 dilution) for 30 min at 37 °C with 5% CO_2_. Cochlear explants were stained with anti-myosin 7a antibody (1:500 dilution). After washing the samples three times with 1% PBS, they were incubated overnight at 4 °C with the corresponding secondary antibody. Then, cochlear explants were stained with the cytosolic fluorescent marker DAPI for 10 min at room temperature. After staining, the samples were observed under a confocal fluorescence microscope (Leica SP8; Leica Microsystems, Germany). Fluorescence intensity was measured by flow cytometry and quantified using FlowJov10.

### 4.9. RNA Isolation and Quantitative Real-Time PCR

The HEI-OC1 cells were treated and then washed three times with PBS. Total RNA was extracted using the RNA isolator Total RNA Extraction Reagent (R401; Vazyme, Shanghai, China). The extracted total RNA was used for the reverse transcription of miRNA and mRNA to obtain cDNA using the miRNA 1st Strand cDNA Synthesis Kit (MR201; Vazyme, Shanghai, China) and PrimerScript Reagent Kit (RR047A; Takara, Kusatsu, Japan), respectively. U6 and β-actin were used as internal controls for miRNA and mRNA, respectively. Real-time PCR was performed using Taq Pro Universal SYBR qPCR Master Mix (Q712; Vazyme, Shanghai, China) for miRNA and SYBR Premix Ex TaqII (RR082A; Takara, Japan) for mRNA on a BIO-RAD instrument, following the manufacturer’s instructions. The primer sequences used in the experiment included GPX4 forward primer: 5′-CAACCAGTTTGGGGAGGCAGGAG-3′, reverse primer: 5′-TAGCACGGCAGGTCCTTCTCTATC-3′; β-actin forward primer: 5′-GTCCCTCACCCTCCCAAAAG-3′, reverse primer: 5′-GCTGCCTCAACACCTCAACCC-3′; miR-182-5p primer: 5′-TTTGGCAATGGTAGAACTCACACCG-3′; and U6 forward primer: 5′-GCTTCGGCAGCACATATACTAA-3′, reverse primer: 5′-AACGCTTCACGAATTTGCGT-3′. The transcript levels of miRNAs and mRNAs were quantified using the 2^−ΔΔCT^ method.

### 4.10. Western Blot Analysis

The cells were washed three times with PBS for 5 min each and then lysed with RIPA cell tissue lysate (Beyotime, Shanghai, China) containing 1% PMSF (Beyotime, Shanghai, China) for 30 min on ice. The lysate was centrifuged at 4 °C and 12,000 rpm for 10 min, and the supernatant was collected to determine the protein concentration using the BCA Protein Assay Kit (Beyotime, Shanghai, China). Protein samples were denatured by adding SDS-PAGE Sample Loading Buffer (Beyotime, Shanghai, China) and heating at 100 °C for 10 min. The proteins were separated on a 12% SDS-PAGE gel and transferred onto polyvinylidene difluoride membranes (Immobilon-P, Millipore, Schaffhausen, Switzerland). The membranes were blocked with 5% nonfat dry milk in TBST for 1 h at room temperature and then incubated with anti-GPX4 antibody (1:1000 dilution; 125066, Abcam, Cambridge, UK), anti-GAPDH (1:1000 dilution; GNI4310-GH, GNI, Tokyo, Japan), or anti-β-actin (1:1000 dilution; GNI4310-BA, GNI, Japan) at 4 °C overnight. After incubation with secondary antibodies for 2 h at room temperature, protein signals were visualized using an ultrasensitive ECL chemiluminescence kit (P0018S, Beyotime, Shanghai, China). ImageJ 2.3.0/1.53S software was used for semi-quantitative analysis to compare the relative expression of proteins.

### 4.11. Dual Luciferase Reporter Gene Assay

To investigate the miRNA–target gene relationship, we conducted a dual luciferase assay. We constructed both a wild-type (WT) and mutated (MUT) mouse GPX4 3′UTR plasmid by cloning the corresponding sequences into the pRL-TK plasmid vector. When cell confluence reached approximately 70%, target cells were transfected with the constructed reporter plasmids using HG transgene reagent (TG-10012, Genomeditech, Shanghai, China). Transfection was carried out for 48 h, after which the reporter gene was detected using the GM-040502A reporter gene detection kit (Genomeditech, Shanghai, China) as per the manufacturer’s protocol. Fluorescence signal intensity was quantified using an enzyme marker (SpectraMax L, MOLECULAR DEVICES, Shanghai, China) and normalized to the sea kidney signal.

### 4.12. ABR Test

To investigate the impact of sevoflurane anesthesia and ferroprost-1 pretreatment on mouse hearing, we conducted auditory brainstem response (ABR) tests. Mice were initially anesthetized with an intraperitoneal injection of 100 mg/kg ketamine and 25 mg/kg xylazine sodium and then maintained at a body temperature of 37 °C on a heating pad. A TDT System III instrument (Tucker Davies Technologies, Gainesville, FL, USA) and SigGenRZ 5.7.6 software were used to test the hearing condition of three group mice at frequencies of 8 kHz, 16 kHz, 24 kHz, and 32 kHz, with intensities ranging from 90 dB to 20 dB in 5 dB increments. Responses were recorded, digitized, and averaged using BioSigRZ software 5.7.6 with 1024 samples per intensity level.

### 4.13. Statistical Analysis

The data were analyzed using GraphPad Prism 9. One-way ANOVA was used for comparisons between multiple groups of data; Bonferroni correction was used to limit the risk of false-positive inferences; and n represents the number of samples in each group. All values were expressed as the mean ± S.E.M. The results were considered statistically significant at *p* < 0.05.

## 5. Conclusions

Firstly, our study demonstrated that sevoflurane exposure could lead to hearing damage and iron overload and lipid peroxide accumulation in auditory hair cells of developing mice, which could be alleviated by the use of ferrostatin-1. Second, the expression of GPX4, a core regulator of ferroptosis, was decreased in auditory hair cells after sevoflurane exposure. miR-182-5p could target and regulate the expression of GPX4 in HEI-OC1 cells. In conclusion, our study demonstrated that inhibition of miR-182-5p upregulated GPX4 expression, thereby attenuating sevoflurane-induced ferroptosis in auditory hair cells.

## Data Availability

The data used in this study and the data analysis are available upon request from the corresponding author.
